# Harnessing peatland rewetting for effective biochar-based carbon dioxide removal

**DOI:** 10.1007/s42773-025-00524-5

**Published:** 2026-01-23

**Authors:** Jennifer M. Rhymes, Niall P. McNamara, Davey L. Jones, Fabrizio Albanito, Chris D. Evans

**Affiliations:** 1 UK Centre for Ecology & Hydrology, Environment Centre Wales, Bangor, LL57 2UW UK; 2 UK Centre for Ecology & Hydrology, Lancaster Environment Centre, Lancaster, LA14 AP UK; 3 School of Environmental and Natural Sciences, Bangor University, Bangor, Gwynedd LL57 2UW UK

**Keywords:** CO_2_ removal, Biochar stability, Negative emissions technology, Peatland restoration, Rewetting

## Abstract

Biochar is widely recognised as a carbon dioxide removal (CDR) technology, but its stability depends on feedstock, pyrolysis conditions, and the soil environment. Current CDR schemes prioritise highly stable biochars to ensure long-term permanence, requiring high pyrolysis temperatures that reduce carbon yield and intensify competition for biomass. This perspective explores potential synergies between two distinct CDR approaches, biochar application and peatland rewetting, where rewetted peatlands could enhance biochar permanence by suppressing microbial decomposition, offering a means to improve both carbon retention and resource efficiency. Using decomposition rate modifiers from biogeochemical models, we estimate biochar stability in rewetted peat and assess its CDR efficiency relative to a counterfactual of high-stability biochar application to dry soils. This perspective suggests that rewetted peatlands significantly reduce biochar carbon losses, particularly for lower-stability biochars, making them more viable for long-term CDR. By allowing greater flexibility in biochar selection, this approach could improve the scalability of biochar deployment while alleviating biomass supply constraints. While challenges such as land-use transitions and methane emissions must be addressed, integrating biochar with peatland rewetting presents a high-impact strategy to optimise the efficiency of biomass-based CDR.

**Graphical Abstract:**

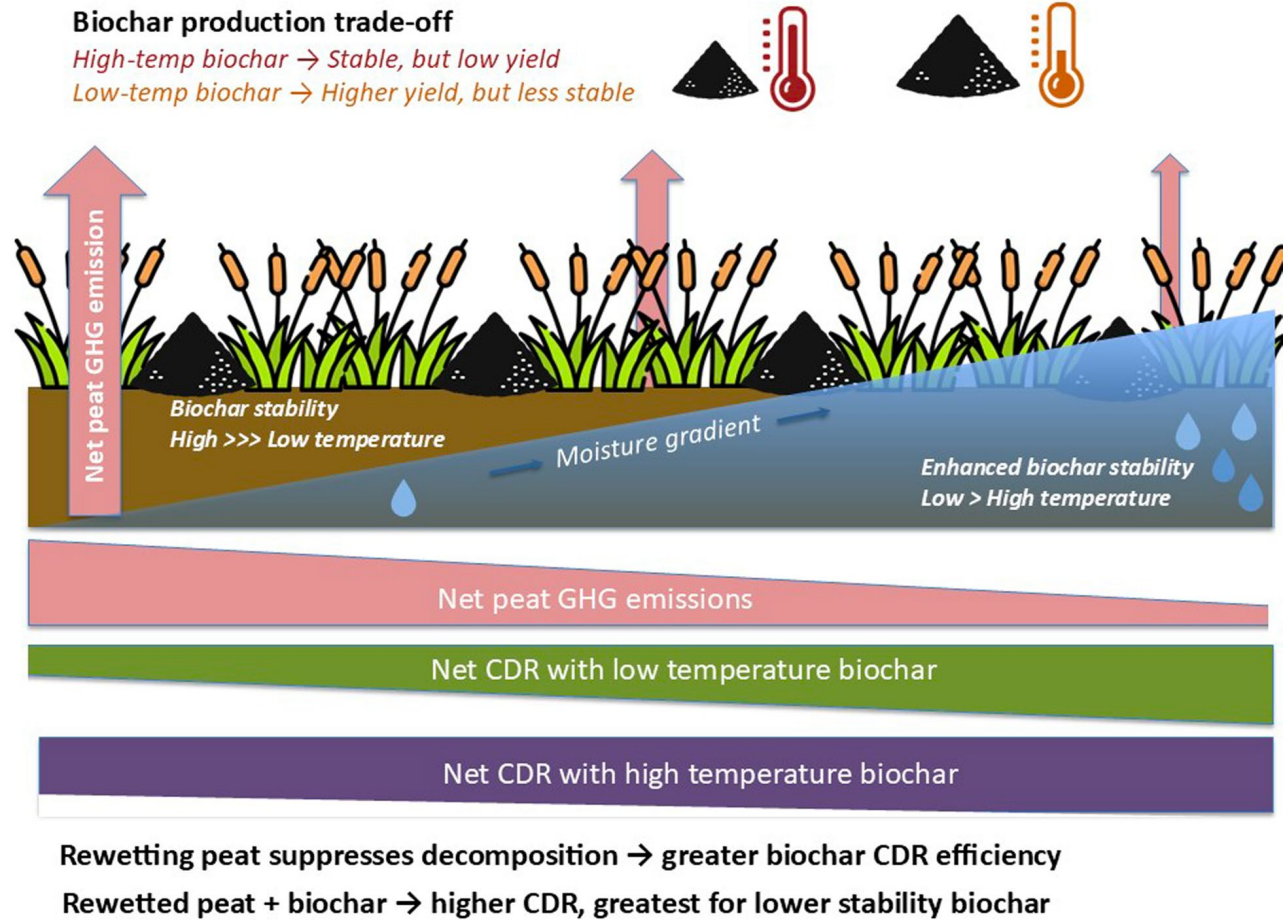

**Supplementary Information:**

The online version contains supplementary material available at 10.1007/s42773-025-00524-5.

## Highlights

- Rewetted peatlands significantly enhance biochar stability, particularly for less stable, higher-yield biochars.
- Lower stability biochars in rewetted environments offer greater CO_2_ removal efficiency.
- Integrating biochar with peatland rewetting creates synergistic climate mitigation benefits despite implementation challenges.

**Supplementary Information:**

The online version contains supplementary material available at 10.1007/s42773-025-00524-5.

## Introduction

Biochar is widely recognised for its carbon dioxide removal (CDR) potential due to its chemically stable structure and ability to persist in soils for centuries (Chiaramonti et al. 2024). However, its stability and persistence depend on its physiochemical properties, which are influenced by feedstock type and pyrolysis conditions (Li et al. 2023) as well as a range of edaphic factors in which it is applied (Wang et al. 2016). While relationships between biochar stability and biochar physiochemical properties are well documented (Li et al. 2023), far less research has effectively examined how soil conditions and processes influence its long-term stability. As a result, efforts to use biochar for CDR primarily focus on highly recalcitrant biochars, which provide greater assurance of long-term carbon storage, regardless of soil conditions. This emphasis is reinforced by voluntary carbon markets, where biochar stability is a key parameter in CDR biochar guidelines and, in some cases, a compulsory requirement for credit eligibility. Standards often specify acceptable thresholds to ensure only highly stable biochars qualify for CDR credits. Producing these highly stable biochars typically requires high pyrolysis temperatures, which significantly reduce the carbon yield from the original biomass feedstock (~ 25–50%), making it a less efficient form of carbon capture (Rodrigues et al. 2023).

Despite their high stability, even highly recalcitrant biochars contain a small proportion of labile organic and inorganic carbon that is expected to mineralise rapidly in soil (Woolf et al. 2021). These carbon losses vary significantly and are often poorly quantified, raising questions about CDR effectiveness. Many schemes assume high rates of biochar persistence regardless of where it is applied, treating its carbon storage as effectively permanent. Furthermore, most biochar applications are made to well-managed aerobic mineral soils used for agriculture, where moisture levels are typically maintained near the optimum for microbial activity. These conditions favour rapid carbon turnover, raising uncertainty about biochar stability in such environments. Given these considerations, targeting soil conditions that supress or completely halt losses via microbial decomposition may be a way to maximise biochar’s CDR potential. Rewetted peatlands present a promising opportunity in this regard.

Rewetting peatlands is also recognised as a nature-based CDR pathway (e.g., Borchers et al. 2024). Globally, peatlands drained for agriculture have become significant greenhouse gas (GHG) sources, contributing approximately 3–4% of global anthropogenic emissions (Leifeld and Menichetti 2018). Rewetting peat limits its O_2_ supply, supressing organic matter decomposition and significantly reducing CO_2_ emissions. As a consequence, abatement of GHG emissions from drained peatlands is a high priority for climate change mitigation, and is included in national strategies of countries ranging from the UK (Climate Change Committee 2024) to Indonesia (Yuwati et al. 2021). However, the potential role of peat rewetting in CDR is more limited; only in cases where rewetting enables carbon sequestration to resume through peat formation does it contribute to carbon removals. Additionally, rewetting can lead to increased methane (CH_4_) emissions, which (although largely representing a return to pre-disturbance conditions, Evans and Gauci 2023) may offset some of its climate benefits. This was highlighted as an implementation risk by The Royal Society’s Greenhouse Gas Removal report, leading to low projected levels of feasible GHG removal from wetland restoration (The Royal Society 2017).

Applying biochar to rewetted peatlands, where anaerobic conditions naturally restrict organic matter decomposition, holds considerable potential to enhance biochar CDR. Biochar decomposition is primarily microbially mediated (Wang et al. 2016) and strongly influenced by soil moisture (Nguyen and Lehmann 2009; Foereid et al. 2011). Higher water saturation reduces O_2_ availability, thereby suppressing microbial activity (Henry 2012). Studies have shown that biochar degradation is significantly lower under flooded conditions, compared to unsaturated conditions (60% of water holding capacity; Nguyen and Lehmann 2009). This suggests that rewetted peatlands could provide an environment where biochar stability is further prolonged, enhancing its viability as a CDR strategy while complementing existing peatland rewetting efforts.

The cost of biochar production is context dependent including consideration of feedstock and labour costs, pyrolysis technological approach (and co-products), all of which vary geographically. Whilst the economics of pyrolysis may optimise for a certain temperature incorporating carbon prices might select for conditions at lower temperatures allowing a higher biochar yield. Targeting rewetted peat soils for biochar application could therefore offer opportunities to use less stable biochars, which are cheaper and more energy efficient to produce. These biochars are generally not favoured for CDR due to their susceptibility to higher carbon losses from microbial decomposition (Al‐Wabel et al. 2018). This reduced stability is associated with lower pyrolysis temperatures (< 400 °C), which decrease the proportion of recalcitrant (stable) aromatic carbon in biochar (Mašek et al. 2013). In this study, we compare biochars across a stability gradient defined by their H/C_org_molar ratios, a widely used proxy for persistence in soil (Schimmelpfennig and Glaser 2012). Biochars with H/C_org_ratios between 0.6 and 0.7 are typically produced at lower pyrolysis temperatures (< 400 °C) which we refer to hereafter as ‘lower-stability’ biochars. While still within the stable classification used by voluntary carbon markets (EBC standards), we use this term in a comparative sense to distinguish them from biochars with H/C_org_ values of 0.1–0.3, produced under higher temperature conditions (> 500 °C).

Consequently, pyrolysis temperatures and H/C_org_ molar ratios are often used as proxies to estimate biochar stability (Leng et al. 2019). In this context, high H/C_org_ molar ratios (> 0.6) are associated with low-temperature (< 400 °C) pyrolysis conditions, while lower H/C_org_molar ratios (0.2–0.6) correspond to high-temperature pyrolysis conditions (Schimmelpfennig and Glaser 2012).

There is a general misconception that greater biochar stability, as indicated by lower H/C_org_ molar ratios inherently translates to higher CDR potential. This view does not fully account for the trade-off between stability and carbon yield. Higher pyrolysis temperatures increase aromaticity and stability, but also result in lower biochar yields as more feedstock carbon is converted into syngas and bio-oils. Although these co-products may contribute to emissions abatement when used as energy sources, they do not offer the permanence associated with sequestered carbon in biochar. From a strict CDR perspective, carbon retained in the solid fraction is key.

Rodrigues et al. (2023) make this argument, and demonstrate that pyrolysis temperatures exceeding 600 °C result in lower biochar yields (relative to the carbon content of the feedstock) and therefore reduced overall CDR efficiency compared to biochar produced at 500–550 °C. Given the limited land area available for biomass production in most countries, the availability of feedstock is generally recognised as a limiting factor for the amount of CDR that could be delivered using biochar (The Royal Society 2017). This limitation is expected to become increasingly acute in the future due to competition for woody biomass from sectors including energy, construction, and green steel manufacture (Woolf et al. 2010). The trade-off between biochar stability and yield exacerbates this limitation, therefore representing a significant constraint on the scalability of biochar deployment for carbon capture.

This conceptual study explores potential synergies between two distinct CDR approaches, biochar application and peatland rewetting, to determine whether their combination could deliver climate benefits exceeding those achieved when implemented individually. In effect, could the introduction of an abiotic constraint on decomposition break the biochar stability versus yield trade-off?

## Conceptual model for biochar permanence in rewetted peat—approach

We extracted data from Rodrigues et al. (2023) using WebPlotDigitizer to obtain the relationship between biochar H/C_org_ molar ratio and the 100-year biochar carbon retention fraction (*Fperm*). To estimate biochar permanence in rewetted peatlands, we adjusted the extracted *Fperm* values using soil moisture modifiers derived from biogeochemical process-based models, including ECOSSE (Smith et al. 2007), Daycent (Parton et al. 1998), and StandCarb (Harmon and Domingo 2001). We used all three models to generate a mean adjusted *Fperm* estimate under rewetted conditions. These models were chosen as they apply empirical soil moisture rate modifiers to account for decomposition rate changes under hydric conditions (see section S1 for further justification).

To quantify the increase in biochar retention under rewetted peatland conditions, we calculated thedifference between rewetted and soils with mineral soils with optimal decomposition rates (we refer to these as reference soils), reflected by *Fperm* values extracted from Rodrigues et al. (2023). The reference soils were chosen as a counterfactual because biochar is most commonly applied to agricultural soils that are aerobic and well-managed, with moisture conditions near the optimum for microbial decomposition, but not waterlogged. These conditions represent typical application environments and provide a realistic baseline for biochar application. The increase in carbon retention from biochar application to rewetted peat soils was calculated by subtracting the adjusted *Fperm* from the original *Fperm* values extracted from Rodrigues et al. (2023) and multiplying the result by 100:

$$Carbon Retention Increase = \left(Adjusted Fperm-Original Fperm\right) \times 100$$

Using the same dataset with adjusted *Fperm* values, we binned the H/C_org_ ratios into two categories: 0.1–0.2 to represent high stability biochars and 0.6–0.7 to represent lower stability biochars. Each bin contained seven observations for 0.1–0.2 and eight for 0.6–0.8 per soil type. We estimated the decay rate constant (*k*) for each observation:

$$k=\frac{-\mathrm{ln}\left(1-Fperm\right)}{100}$$

These decay rate constants were then used to fit an exponential decay model describing biochar carbon loss over 100 years for High and Lower Stability biochars per soil type.

Drawing on another dataset from Rodrigues et al. (2023) with biochar carbon yields, this is the share of C fixed in the biochar relative to the amount of C in the feedstock (wt%) used to produce the biochar, corresponding to *Fperm* values and H/C_org_ ratios for different feedstocks (*n* = 12). Here we adjusted *Fperm* values as earlier to ensure we have values that reflect rewetted peatlands. We then used the same approach as Rodrigues et al. (2023) to calculate CDR potential with the following equation:

$$CDR Potential=Fperm\times Biochar Carbon Yield$$

We determined the increase in CDR potential for targeting rewetted peatland by calculating the effective difference between CDR potentials estimated for rewetted peat soils (Adjusted CDR) and soils with optimal decomposition rates (Original CDR), using the following:

$$Effective CDR change \left(\mathrm{\%}\right) = Adjusted CDR - Original CDR$$

To support interpretation, we used the adjusted F_perm_ and biochar yield values to calculate absolute carbon flows per 1 tonne of feedstock carbon. Pyrolysis loss was derived from (1 – biochar yield), while biochar carbon loss and CDR were calculated using the decay model and CDR estimation approach described above.

### Moisture rate modifier justification

The three process-based models considered in this study—ECOSSE, Daycent, and StandCarb—were selected based on their ability to simulate SOC turnover rates under varying moisture conditions (Sierra et al. 2015). Each model incorporates empirical soil moisture rate modifiers to adjust decomposition rates, which we applied to estimate biochar stability under rewetted conditions. Soil decomposition rates peak under optimal moisture conditions, where oxygen availability and decomposable substrates are maximised (Skopp et al. 1990; Moyano et al. 2013; Sierra et al. 2015). In contrast, decomposition slows in dry soils due to hydraulic constraints on substrate diffusion and microbial activity, and in waterlogged soils due to oxygen limitation (Manzoni and Katul 2014; Schimel 2018). By incorporating these constraints into our adjusted *Fperm* values, we generated a mean estimate of biochar stability in rewetted peatlands, ensuring that the applied decomposition rates reflect realistic environmental conditions.

## Optimising biochar CDR by targeting rewetted peatlands

Our findings demonstrate that biochar carbon losses over 100 years in rewetted peat soils are significantly lower than current estimates based on aerobic mineral soils with optimal decomposition conditions (hereafter referred to as reference soils). This trend holds across biochar stability classes, highlighting the potential of rewetted peatlands to enhance biochar CDR efficiency. Notably, the retention benefits are most pronounced for biochars with lower stability, which are more susceptible to microbial decomposition under typical soil conditions.

**Fig. 1:**
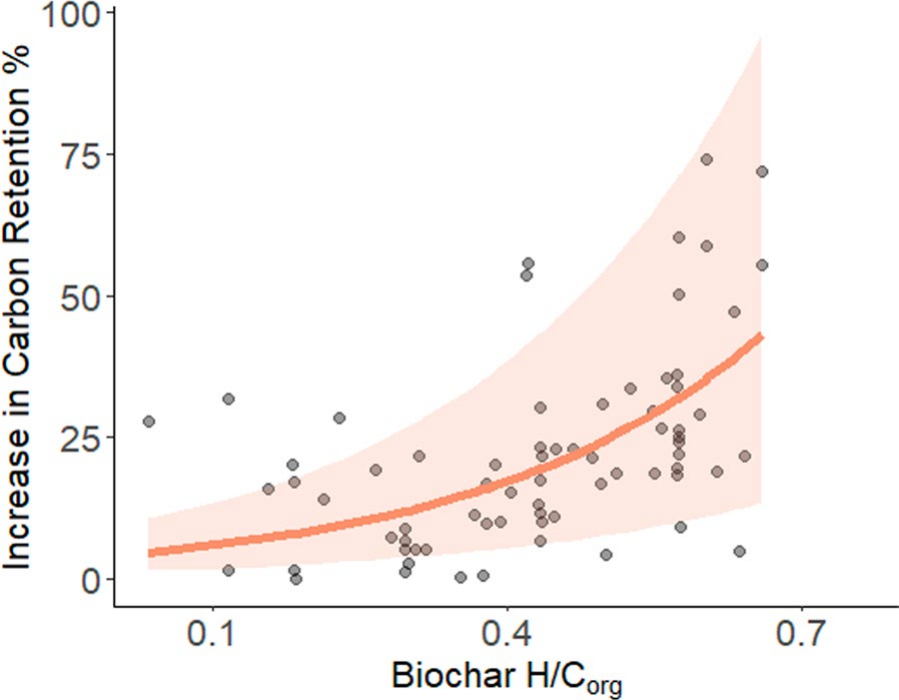
Relationship between biochar H/C_org_ molar ratio (a proxy for biochar stability, high stability to the left and lower stability to the right) and the increase in biochar carbon retention over a 100-year period when applied to rewetted peat soils, relative to reference soils. Points represent calculated carbon retention increase. The fitted exponential model (solid line) represents the predicted increase in retention, while the shaded area indicates the 95% confidence interval derived from bootstrapped model estimates

When losses are expressed as a percentage of total biochar, rewetted peat soils enhance biochar carbon retention (Fig. 1) by approximately 5% for highly stable biochars (H/C_org_ = 0.1) and 40% for less stable biochars (H/C_org_ = 0.7). This effect is more pronounced for the lower stability biochars, because they contain a greater proportion of labile carbon that would otherwise be highly susceptible to microbial decomposition (~ 67% loss under optimal conditions). In contrast, highly stable biochars already experience relatively low losses under optimal conditions (~ 20%), meaning the enhancement is less pronounced.

**Fig. 2:**
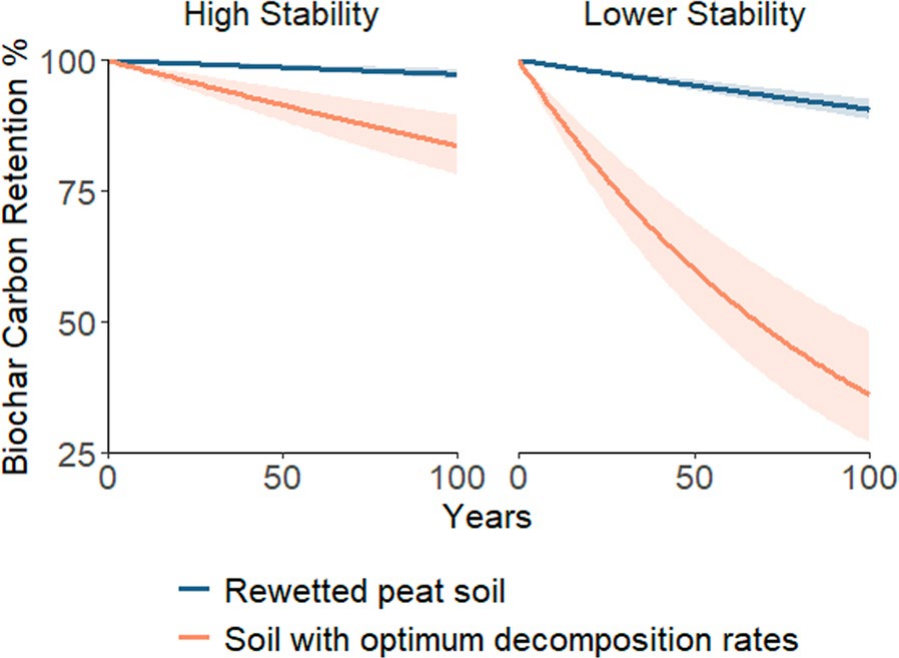
Modelled decay of biochar carbon over 100 years in rewetted peat soils (blue) and agricultural soils with optimal decomposition rates (orange). The decay curves represent the fraction of biochar carbon retained in soil over time, with separate panels for biochars of high stability (H/C_org_ = 0.1–0.2) and lower stability (H/C_org_ = 0.6–0.7). Solid lines indicate the mean decay trajectory, while the shaded regions represent the 95% confidence intervals derived from the standard error of decay rates

Modelled decay trajectories further support these findings, demonstrating how biochar retention changes over time in rewetted peatlands compared to reference soils (Fig. 2). Over the 100-year period, highly stable biochars (H/C_org_ = 0.1–0.2) show minimal losses under both conditions, though retention is still enhanced in rewetted peat soils. In contrast, lower-stability biochars (H/C_org_ = 0.6–0.7) show a more pronounced divergence. In the early years following application, retention differences between soil types are modest, but as time progresses, the effects of rewetting become increasingly apparent, particularly for biochars with a higher labile carbon content. This highlights the role of rewetting in extending biochar persistence over time, particularly for biochars more susceptible to microbial decomposition.

It is important to note that Fig. 2 is a conceptual representation of these trends, based on binned categories of biochar stability (H/C_org_ = 0.1–0.2 for high stability and 0.6–0.7 for lower stability). This differs from Fig. 1, which incorporates the full dataset and provides a continuous relationship between biochar stability and retention. While Fig. 2 simplifies the comparison between biochar types, Fig. 1 presents a more detailed data-driven trend across all observations.

**Fig. 3:**
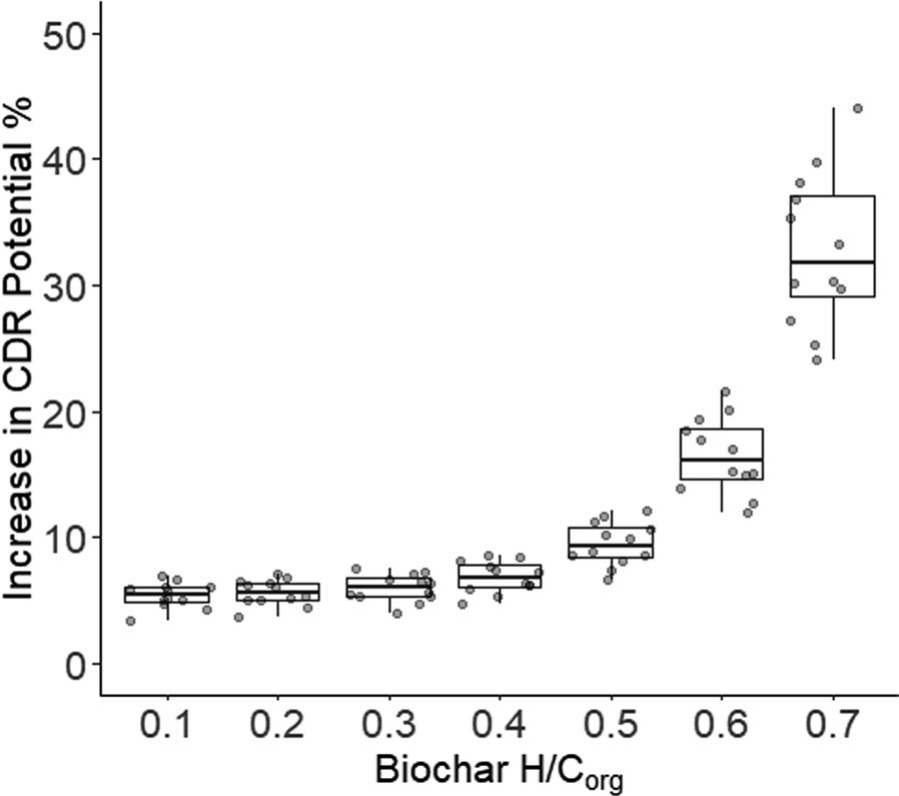
Effective carbon dioxide removal (CDR) potential expressed as the percentage of the original carbon content of the feedstock retained after biochar production and application to rewetted peat soils, relative to aerobic agricultural soils with optimal decomposition rates for a range of biochar H/C_org_ molar ratio categories. Boxplots illustrate the effective enhancement in biochar CDR. Each box represents the interquartile range (25th to 75th percentile), with the horizontal line inside the box indicating the median value. The error bars represent the 10th and 90th percentiles. Points represent individual data observations to demonstrate distributions

Beyond biochar retention, we assessed biochar CDR efficiency in rewetted peat soils (Fig. 3), which accounts for the retention of the original carbon feedstock. Boxplots illustrate that application of biochar with H/C_org_ = 0.7 to rewetted peat achieves a 30% increase in CDR efficiency relative to aerobic agricultural soils with optimal decomposition rates, whilst more stable biochars with H/C_org_ = 0.1–0.3 showed only a 5% improvement.

To aid interpretation of these outcomes, we provide a summary of the modelled carbon flows for two representative biochars in Table 1. The table illustrates the fate of carbon from 1 tonne of feedstock carbon, partitioned into pyrolysis losses, post-application decomposition, and resulting CDR after 100 years. This clearly highlights how rewetted peat environments enhance long-term carbon retention, particularly for lower-stability biochars.

**Table 1:** Carbon flows modelled for 1 tonne of carbon input in the feedstock, comparing high- (H/C_org_ = 0.1) and lower-stability (H/C_org_ = 0.7) biochars applied to either dry reference soils or rewetted peat

H/C_org_ molar ratio	Soil type	Biochar C retained (kg)	C loss during production (kg)	Biochar C loss in soil (kg)	CDR (kg)
0.1	Reference soil	448	552	65	383
0.1	Rewetted peat	448	552	11	437
0.7	Reference soil	546	454	397	149
0.7	Rewetted peat	546	454	68	478

Values represent the mass of carbon (kg) retained after pyrolysis, loss during production, decomposed in soil over 100 years, and the remaining carbon assumed sequestered as carbon dioxide removal (CDR) over 100 years

Our analysis focuses on the effective change in CDR efficiency between rewetted peatlands and reference soils (Fig. 3). On this more holistic metric, the lowest stability biochars explored in this study (H/C_org_ = 0.7) offered the highest relative improvement in CDR potential in rewetted peatlands, with a 33% improvement relative to the reference soils, whilst still offering a 5% increase for the higher stability biochars (H/C_org_ = 0.1–0.3). This is notably different from the optimal H/C_org_ ratio of 0.4 identified by Rodrigues et al. (2023). The reason for this shift is that although lower-stability biochars undergo greater carbon losses when in the soil, they also retain a larger fraction of the total feedstock carbon due to higher biochar yields during pyrolysis, leading to a net increase in long-term CDR.

Based on this analysis, we propose that the application of ‘low-stability’ biochars will lead to higher CDR than that of ‘high-stability’ biochars when viewed from a whole-system perspective. These seemingly counterintuitive findings suggest the potential for a fundamental shift in how—and where—biochar should be deployed for CDR. Our findings also challenge the assumption that only highly stable biochars should be considered suitable for climate mitigation.

Currently, however, the commercial sale of biochar is largely driven by its agronomic benefits, including improvements to soil structure, soil pH, water retention, and nutrient availability. Due to high costs and regulatory thresholds (e.g., 1 t ha⁻^1^ y⁻^1^ in the UK; Environment Agency), farmers can only apply biochar at low rates. From a CDR perspective, these low-dose applications across large areas provide minimal per-hectare carbon sequestration benefits, and are also inefficient in terms of time, fuel costs, and associated CO_2_ emissions of application. This makes widespread agricultural biochar applications inefficient for CDR, while also posing significant challenges for measurement, reporting, and verification (MRV). Some studies indicate that biochar is a poor investment for farmers, particularly for cereal production (Dickinson et al. 2015), further limiting its uptake in conventional agriculture. In this context, rewetted peatlands provide an alternative application pathway with the potential for much higher application rates at smaller spatial scales. Over time biochar applied in surface layers is expected to move deeper into the peat matrix (Leifeld et al. 2007). Subject to regulatory approval, such an approach has significant potential to improve biochar CDR efficiency. This is particularly relevant for peatland rewetting projects with one-off capital applications or as part of peatland infill strategies (we discuss these further in Sect. 5).

Rather than focusing exclusively on maximising biochar stability, these findings support a more strategic approach—one that balances production efficiency with long-term CDR potential. This approach could help scale biochar deployment while ensuring that biomass resources are used as effectively as possible in the wider CDR landscape.

## Model uncertainties and limitations

A key uncertainty in this study is the assumption that the two-pool exponential decay methodology, widely used in policy guidelines and national CDR accounting, accurately predicts biochar permanence over a 100-year timescale (Woolf et al. 2021; Rodrigues et al. 2023). These models divide biochar carbon into labile (C-pool_1_) and recalcitrant (C-pool_2_) fractions, assigning a higher decay rate to C-pool_1_. A recent review of this methodology suggests that this approach may overestimate the decay rate of the largely inert recalcitrant fraction (Sanei et al. 2025).

In the context of this study, biochar permanence in rewetted peatlands may be underestimated, potentially leading to lower reported CDR gains for highly stable biochars. However, even if this is the case, their application would still enhance overall CDR potential. For lower-stability biochars, which contain a higher proportion of labile carbon (C-pool_1_), existing models more accurately capture their decay dynamics, meaning our estimated CDR gains from targeting rewetted peatlands remain applicable. Thus, even if current models overestimate biochar decomposition rates, our findings should still hold, whereby rewetted peatlands create conditions that enhance biochar stability and improve CDR potential compared to drier soil environments.

Our study focuses exclusively on biochars with H/C_org_ ratios < 0.7 due to the limited availability of data for higher ratio biochars. This threshold aligns with certification standards set by the International Biochar Initiative (IBI) and the European Biochar Certificate (EBC), which define biochar eligibility for carbon markets. However, other carbonised materials, such as those produced by torrefaction, fall outside of this classification because of their higher H/C_org_ ratios (despite often being referred to as biochar). While these materials are ineligible for biochar certification, they may still have potential applications in rewetted peatlands, particularly in cases where feedstock carbon retention from increased biochar yields outweigh total biochar carbon losses.

Furthermore, the main limitation of using a 100-year timescale is that it underestimates biochar’s true carbon storage potential, as multiple studies have shown that a large share of pyrogenic carbon persists for over 1,000 years (Schmidt et al. 2022). However, we adopt this timeframe because it aligns with the reporting period used by Rodrigues et al. (2023), one of the few studies linking biochar carbon yield to modelled retention, and reflects current policy-relevant benchmarks for long-term carbon removal.

## Practicalities and mechanisms for combined biochar and peatland CDR

There is a growing global agenda to rewet previously drained peatlands for climate mitigation and ecosystem restoration. The Paris Agreement outlines a commitment to rewet 50 million hectares of drained peatlands to help achieve carbon neutrality by 2050–2070 (IPCC 2018), averaging over one million hectares per year. This large-scale rewetting effort highlights the scalability potential for integrating biochar-based CDR with peatland rewetting efforts.

Previously drained peatlands can be rewetted for various purposes, with or without changes in land use (Tanneberger et al. 2021). Building on this, we identify several potential strategies for biochar application in rewetted peatlands, considering both the intended outcomes and the methods of application, such as soil incorporation versus surface spreading (Roofchaee et al. 2024). These strategies include:

1. **One-off capital application**—A large-scale, single application at the 'capital works' stage of a restoration project, providing an initial carbon input to the rewetted landscape.
2. **Regular incorporation in paludiculture**—Paludiculture is a form of wet agriculture that involves cultivating crops in waterlogged conditions. Here, biochar could be incorporated periodically as part of standard land management practice, such as during tillage, crop establishment, or harvesting activities. This approach aligns with ongoing farm activities and provides the potential for repeated inputs of biochar over time.
3. **Periodic surface application in conservation areas**—For rewetted peatlands designated for conservation, where minimal disturbance is preferred, surface spreading may be the most suitable approach.
4. **Biochar for peatland infill**—This approach involves depositing biochar into areas where a large volume of soil has historically been lost such as former peat extraction sites, as well as areas being restored after a long period of agricultural drainage, which in some regions has led to several metres of peat subsidence, leaving the ground surface below sea or river levels (REF). This approach would be similar to the existing practice of using conservation waste biomass for infill, and could provide an alternative method for land restoration.

It is crucial to understand how these application methods could impact a biochar CDR efficiency on peat. For example, surface spreading is likely to be less effective for CDR given that the biochar is exposed to the atmosphere, leading to higher oxidation rates. Additionally, this method may increase susceptibility to losses from water and wind erosion (Xiao et al. 2016).

For biochar application to rewetted peatlands to be recognised as a credible CDR strategy, robust MRV will be essential. A practical approach may involve combining biochar-specific MRV with existing peatland MRV frameworks, such as those established under the Peatland Code (IUCN 2022). For example, regular water table depth monitoring, which is already used to verify rewetting success for peat preservation could also serve as a proxy for the saturated conditions required to suppress biochar decomposition and ensure long-term carbon storage.

## Potential co-benefits of biochar application to rewetted peatlands

Methane emissions have been identified as a constraint on the level of net GHG removal that can be achieved through peatland rewetting (The Royal Society 2017). There is some evidence that biochar can reduce methane emissions in waterlogged soils, in this instance paddy soils (Jeffery et al. 2016), but other studies have shown little effect (Song et al. 2016) or even opposing results (Cong et al. 2018).

Recent studies by Jeewani et al. (2025b, a) investigated the effects of biochar application in peat soils under raised water table conditions which suggest that biochar application could also suppress both peat decomposition and methane emissions. If replicated at larger scales this would significantly enhance the overall climate mitigation benefits of biochar application to rewetted peatlands, help to overcome the CO_2_-methane trade-off whilst accelerating peat formation.

Biochar has also been shown to improve crop yields in waterlogged soils, such as paddy rice fields (Jiang et al. 2024), which could enhance the financial viability of farming on rewetted peat soils. This co-benefit is particularly important given that paludiculture is currently considered financially unviable without substantial agricultural subsidies (e.g., de Jong et al. 2021).

## Potential risks of biochar application to rewetted peatlands

Biochar application to soils is generally considered safe and typically does not pose toxicity risks (Godlewska et al. 2021), particularly given adherence to biochar regulatory standards (e.g., EBC and IBI). However, our study suggests that optimal CDR per unit of feedstock could be achieved with less stable biochars, such as torrefied biochar (Amalina et al. 2022). Although these biochars may not meet existing stability thresholds, they can still comply with other environmental standards, such as heavy metal content (which is a function of the feedstock metal content) and polycyclic aromatic hydrocarbon (PAH) limits. Moreover, it is generally accepted that biochar produced at high temperatures tends to have higher PAH concentrations (Shah et al. 2023). Overall, the application of biochar to rewetted peatlands may require adjusted regulatory standards specific to this context.

A key consideration is that lower-stability biochars have a lower carbon content per unit mass than highly stable biochars, which necessitates higher application rates to achieve the same carbon input. While increased biochar application could enhance CDR potential, it may also introduce risks. Reports suggest that high biochar loading can lead to undesirable agronomic effects, such as reduced crop root length and altered soil conditions (Xiang et al. 2021), which could have unintended consequences depending on soil type and crop response. The use of alkaline biochars at high doses may also significantly alter soil pH, with cascading effects on microbial communities (Ding et al. 2016), and dissolved organic carbon (DOC) dynamics.

Quantitatively constraining application rates is therefore important to avoid unintended risks. In a meta-analysis, Liu et al. (2013) found that crop productivity tended to decline significantly at application rates above 40 t ha⁻^1^, with limited yield gains beyond 10 t ha^−1^. While CDR frameworks often express application in t C ha⁻^1^, physical impacts are more closely tied to total mass, making t ha⁻^1^ a more relevant basis for defining safe limits. Existing agronomic thresholds are not designed for high-rate CDR applications and do not account for broader environmental or system-level risks. New, context-specific thresholds will therefore need to be developed through robust, evidence-based assessments. In addition to agronomic and environmental considerations, feedstock availability and pyrolysis yield also place practical limits on how much biochar can realistically be produced and applied per hectare, particularly at larger scales.Beyond application rates, long-term land use stability is another crucial factor. While low-stability biochars in rewetted peatlands may provide optimal CDR efficiency, any subsequent changes in land use that revert to drainage practices could lead to significant biochar and soil carbon losses. As with most land-based CDR schemes ensuring a long-term commitment from landowners through incentivised schemes could help mitigate this risk and must be considered prior to large-scale roll-out.

Environmental variability further complicates biochar permanence. Peatland water tables can fluctuate due to seasonal droughts and other environmental factors, even with effective water management. These drying and rewetting cycles could accelerate biochar decomposition and biochar loss. However, biochar’s ability to enhance soil water retention (Edeh et al. 2020) may help mitigate these effects and could help mitigate peat carbon losses that would otherwise occur due to oxidation, benefiting the overall carbon balance of these systems. Moreover, lower temperature biochar may enhance water retention in rewetted peatlands due to its hydrophilic properties. In contrast, biochar produced at higher temperatures tends to be hydrophobic (Tomczyk et al. 2020), potentially increasing the risk of biochar flotation and offsite transport. This risk is less relevant for peatland infill or one-off capital applications discussed in Sect. 5, as these biochars are applied at deeper depths that are permanently waterlogged, offering assurance for permanent and enhanced CDR. Biochar application to previously pump-drained agricultural landscapes could also offer a relatively high degree of permanence, insofar as these areas will remain waterlogged unless active measures are taken to reinstate pumping.

## Conclusions

This study demonstrates that biochar application to rewetted peatlands could enhance biochar CDR potential by reducing biochar decomposition rates and potentially accelerating native peat formation. This is particularly pronounced for lower-stability biochars, which retain more of the original feedstock carbon than high-stability biochars. This challenges the assumption that only highly stable biochars should be prioritised for CDR and suggests that applying lower-stability biochar to rewetted peatlands could more efficiently transfer carbon fixed via biomass production into stable long-term storage.

Given the constraints on CDR imposed by limited land availability and competition for woody biomass from the energy (including BECCS) and green steel sectors, biochar application to rewetted peatlands could effectively raise the current limits on the amount of CDR that can be achieved using biochar. However, current carbon market standards favour highly stable biochars, which require high pyrolysis temperatures, reducing biochar yield and increasing competition for biomass resources In the UK, regulations currently restrict biochar application to non-waterlogged soils and set a maximum rate of 1 t ha⁻^1^. While these limits are appropriate for conventional agricultural use, they present a significant barrier to CDR-focused applications. In parallel, Verra, a leading global standard body, currently prohibits biochar application to wetlands.

Realising the CDR potential of biochar application to rewetted peatlands will require voluntary carbon market standards to explicitly recognise the enhanced permanence provided by saturated peat soils. Within this context, the use of lower-stability biochars should also be reconsidered due to their higher CDR potential. This would expand the portfolio of viable biochars for CDR and improve biomass-use efficiency. At the same time, it will be important to ensure that any risks associated with biochar application to peatlands are quantified and minimised, and that measures are put in place to ensure effectiveness and permanence. Provided that these challenges can be overcome, we argue that biochar application to rewetted peatlands could significantly augment national and international efforts to maximise CDR and thereby achieve net zero greenhouse gas emissions.

## Supplementary Information

Additional file 1.

## Data Availability

The datasets used derived from Rodrigues et al. 2023 and are available upon request.
