# Supplementary material for: Harnessing peatland rewetting for effective biochar-based carbon dioxide removal
Source: Biochar. 2026 Jan 23;8(1):16. doi: 10.1007/s42773-025-00524-5 (PMC12827367; doi:10.1007/s42773-025-00524-5)
Supplement: Supplementary file 1 — Additional file 1. [file 42773_2025_524_MOESM1_ESM.docx]

**Supplementary Information**

## **S1 Methods**

### **S1.1 Biochar permanence estimations**

Woolf et al. (2021) proposed one methodology to estimate carbon sequestration for biochar application by which the fraction of biochar-carbon remaining in soil after 100 years (*F_perm_*) is estimated as a function of H/C_org_ or pyrolysis temperature; where *F_perm_* is estimated through fitting to a biochar two carbon pool exponential decay model, representing labile and stable fractions of the biochar that vary in degradation rates (Rodrigues et al., 2023; Wang et al., 2016; Woolf et al., 2021).

We used a regression that estimates *F_perm_* as a function of the H/C_org_ molar ratio of the biochar (Rodrigues et al., 2023) to extract *F_perm_* values across a range of H/C_org_ ratios from 0.1 to 0.7 in increments of 0.1:

*F_perm_* = 0.854 – 4.933 × H/C_org_^5.9998^ (Kandel et al. 2020)

To explore the implications of soil water on the biological stability of biochar (*F_perm_*), we applied the moisture modifiers (*m*_w_) used in the Daycent, ECOSSE, and Standcarb models (Fig. S1). Considering ECOSSE as an example, in the functional forms of the moisture modifier (*m*_w_), decomposition rates are modified for available water content values (ψ_c_) ranging from wilting point (ψ_0_) to field capacity (ψ_f_), and from field capacity to saturated available water levels (ψ_s_). In Figure S1, moisture functions of the three models were normalized in such a way that the input moisture values are all in a range from 0 to 1. In most cases, this transformation consisted in expressing the soil moisture metric (e.g., volumetric water content) relative to its maximum value, which is generally dictated by soil porosity. At ψ_0_ (roughly corresponding to a water tension of -1500 kPa, or to 0 mm of plant-available water; i.e. permanent wilting point) soil carbon decomposition is inhibited and *m*_w_ has a value of 0.2. Between ψ_0_ and a water tension of -100 kPa (ψ_i_) (roughly corresponding to 20 mm of available water or field capacity), *m_w_* linearly increases reaching its maximum value of 1. Above ψ_i_, *m*_w_ is maintained at saturation level until the soil available water content reaches field capacity (ψ_f_) (i.e. a water tension of -5 kPa). This means that between ψ_i_ and ψ_f_ soil carbon decomposition is not inhibited as the available soil water content is assumed to be at optimum level for autotrophic and heterotrophic activity (approximately 40% in clay soil). Above ψ_f_ a linear reduction is applied to *m*_w_ which reaches the minimum rate of 0.2 at ψ_s_.


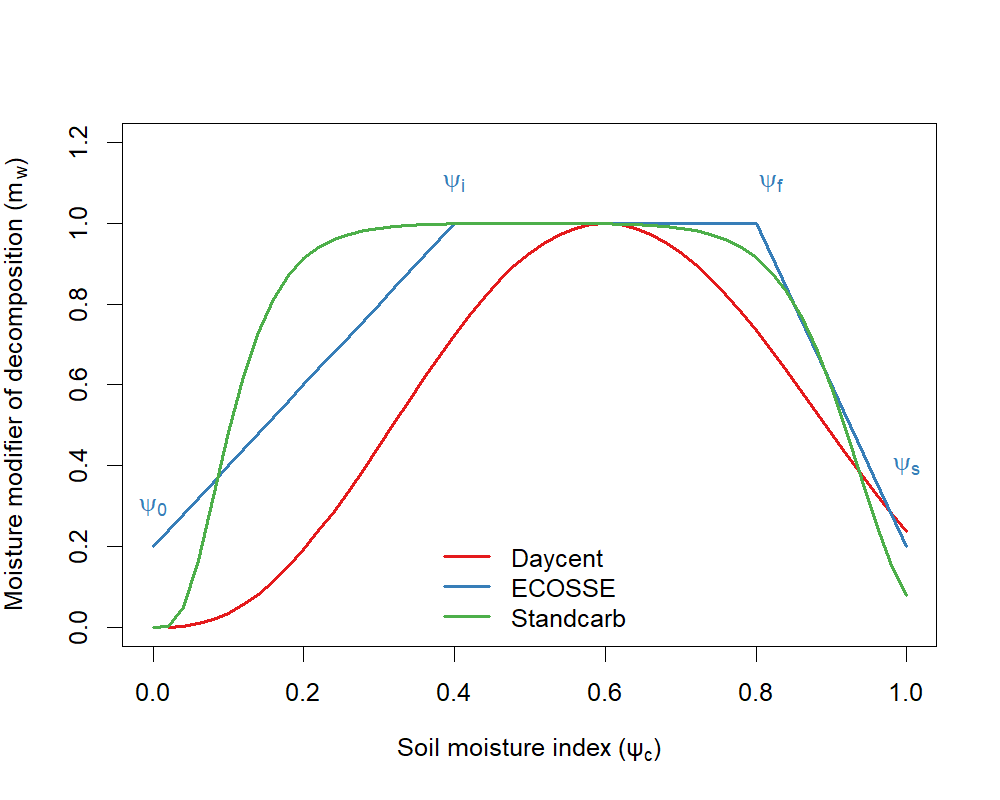


**Fig. S1.** Moisture response functions from the soil carbon turnover models ECOSSE, Daycent, and Standcarb models. Modified from Sierra et al. (2015).

## **S1.2 Model uncertainties**

In general, all models agree that at optimum soil moisture conditions organic matter decomposition rates are “maximum” “and that it is reduced at soil moisture lower than optimum conditions. However, the functions used to represent the effects of soil moisture on SOM decomposition vary depending on the information used in the development of the individual models and the aims and focus of the models (which is normally on aerobic agricultural or forest soils with mineral subsoils). In contrast to the three models used in this study, other models may use moisture modifiers with hump-shaped forms where heterotrophic respiration is controlled by substrate diffusion and oxygen limitation at low and high moisture conditions, respectively (Evans et al., 2022). Such hump-shaped relationships, however, are less commonly observed experimentally than linear or saturating functions (Evans et al. 2022, and references therein). An additional uncertainty arises from the use of single soil moisture modifiers for all ecosystems. The underlying model assumption of using one curve is that the physiology of soil microbial communities is similar, regardless of biome or climate history. By assigning a single moisture response function to all soils, biogeochemical models assume that soil microbes across different ecosystems have the same environmental dependencies. However, this assumption is not supported by some recent studies showing that land use and climate history may affect microbial community function (Evans et al., 2022; Jones et al., 2018, and references therein).

**References**

Evans, S., Steven, A., & Hawkes, C. (2022). Microbes, memory and moisture: Predicting microbial moisture responses and their impact on carbon cycling. *Functional Ecology*, *36*(6), 1430–1441.

Jones, D. L., Hill, P. W., Smith, A. R., Farrell, M., Ge, T., Banning, N. C., & Murphy, D. V. (2018). Role of substrate supply on microbial carbon use efficiency and its role in interpreting soil microbial community-level physiological profiles (CLPP). *Soil Biology and Biochemistry*, *123*, 1–6.

Rodrigues, L., Budai, A., Elsgaard, L., Hardy, B., Keel, S. G., Mondini, C., Plaza, C., & Leifeld, J. (2023). The importance of biochar quality and pyrolysis yield for soil carbon sequestration in practice. *European Journal of Soil Science*, *74*(4), e13396. https://doi.org/https://doi.org/10.1111/ejss.13396

Sierra, C. A., Trumbore, S. E., Davidson, E. A., Vicca, S., & Janssens, I. (2015). Sensitivity of decomposition rates of soil organic matter with respect to simultaneous changes in temperature and moisture. *Journal of Advances in Modeling Earth Systems*, *7*(1), 335–356.

Wang, J., Xiong, Z., & Kuzyakov, Y. (2016). Biochar stability in soil: meta‐analysis of decomposition and priming effects. *Gcb Bioenergy*, *8*(3), 512–523. https://doi.org/https://doi.org/10.1111/gcbb.12266

Woolf, D., Lehmann, J., Ogle, S., Kishimoto-Mo, A. W., McConkey, B., & Baldock, J. (2021). Greenhouse gas inventory model for biochar additions to soil. *Environmental Science & Technology*, *55*(21), 14795–14805. https://doi.org/10.1021/acs.est.1c02425
